# Efficacy and safety of ciprofol versus propofol for induction and maintenance of general anesthesia: a systematic review and meta-analysis

**DOI:** 10.1186/s44158-024-00160-8

**Published:** 2024-04-11

**Authors:** Muhammad Hudaib, Hurais Malik, Syeda Javeria Zakir, Samra Rabbani, Dhanushan Gnanendran, Abdul Rehman Shah Syed, Noor Fatima Suri, Javeria Khan, Arham Iqbal, Nowal Hussain, Muhammad Abdullah, Satesh Kumar, Mahima Khatri, Giustino Varrassi

**Affiliations:** 1 Fazaia Ruth Pfau Medical College, Karachi, Pakistan; 2 Dow University of Health Sciences, Karachi, Pakistan; 3 York and Scarborough Teaching Hospital, NHS Foundation Trust, York, UK; 4 CMH Lahore Medical College and Institute of Dentistry, Lahore, Pakistan; 5 Shaheed Mohtarma Benazir Bhutto Medical College, Karachi, Pakistan; 6 Paolo Procacci Foundation (PPF), Rome, Italy

**Keywords:** Analgesia, Anesthesia induction, Ciprofol, General anesthesia, Propofol, Day surgery

## Abstract

**Background:**

Propofol has been the gold standard for anesthesia induction and maintenance due to its rapid onset and favorable pharmacokinetic properties. However, the search for alternative agents with improved safety and efficacy has led to the emergence of ciprofol (HSK3486), a structural analog of propofol. This systematic review and meta-analysis aim to comprehensively assess the safety and efficacy of ciprofol compared to propofol for anesthesia induction and maintenance in adult patients undergoing surgical procedures.

**Methods:**

This study included only double-arm RCTs in which participants were aged eighteen or older undergoing surgery. For the statistical analysis of the extracted data, we employed RevMan 5.4.1.

**Results:**

Ciprofol demonstrated a promising trend of higher anesthesiologists’ satisfaction during the induction phase (MD 0.14, 95%, CI − 0.28 to 0.56, *p* = 0.51), whereas Propofol was favored during maintenance. Propofol also exhibited advantages with a shorter time to successful anesthesia induction (MD 0.08 min, 95% CI 0.00 to 0.15, *p* = 0.04), and quicker attainment of full alertness (MD 0.11 min, 95% CI − 1.29 to 1.52, *p* = 0.87), suggesting its efficiency in clinical practice. Importantly, there were no significant disparities in the success rate of anesthesia.

**Conclusion:**

Both ciprofol and propofol demonstrate comparable efficacy and safety for anesthesia induction and maintenance in adult patients undergoing surgery. While propofol provides a faster onset of induction, ciprofol exhibits advantages in terms of pain management. Clinicians should consider these findings when selecting anesthetic agents, and tailoring choices to individual patient needs and clinical scenarios.

**Supplementary Information:**

The online version contains supplementary material available at 10.1186/s44158-024-00160-8.

## What is already known about this subject?

Propofol has been the gold standard for anesthesia induction and maintenance due to its rapid onset and favorable pharmacokinetic properties.

## What this study adds

- This systematic review and meta-analysis comprehensively assessed the safety and efficacy of ciprofol compared to propofol for anesthesia induction and maintenance in adult patients undergoing surgical procedures.
- Propofol and Ciprofol exhibited similar efficacy and safety profiles. Nevertheless, Propofol achieved general anesthesia induction more rapidly.
- With Ciprofol there was a reduced incidence of pain at the injection site.

## Introduction

General anesthesia is a cornerstone of modern medical practice, designed to achieve the vital goals of amnesia, unconsciousness (hypnosis), and immobilization during surgical procedures. These objectives are met through the use of general anesthetics, which exhibit the remarkable ability to reversibly induce these therapeutic effects [1, 2]. Among the diverse classes of anesthetic agents, both volatile and intravenous anesthetics play pivotal roles in ensuring reliable and effective anesthesia.

Propofol, a potent γ-aminobutyric acid (GABA) receptor agonist, stands as a testament to the success of intravenous anesthetics over the past three decades [3, 4]. Its favorable pharmacokinetic (PK) and pharmacodynamic (PD) properties have propelled it to the forefront of anesthesia practice. Known for its rapid and consistent induction, minimal excitation phenomena, short context-sensitive time, rapid terminal half-life, and low incidence of postoperative nausea and vomiting, propofol has become a cornerstone of anesthesia induction and maintenance [3]. Nevertheless, even with its exceptional attributes, propofol is not without limitations, which include injection pain, hypotension, respiratory depression leading to apnea, and the potential for the development of intensive care unit (ICU) syndrome [5–7]. It continues to serve as the gold standard against which newer agents are benchmarked. One of these agents is ciprofol (HSK3486).

In recent years, the field of anesthesiology has experienced a surge in the exploration of novel agents for both induction and maintenance of general anesthesia. Among these, ciprofol has emerged as a promising contender, boasting claims of enhanced safety and efficacy when compared to traditional agents. First reported in 2017, ciprofol represents a structural analog of propofol, incorporating an R-chiral center and a cyclopropyl group that imparts improved pharmacological and physicochemical properties. These enhancements render ciprofol more potent than propofol and, notably, less painful upon injection [8, 9]. A phase 1 trial demonstrated the safety of ciprofol at doses ranging from 0.15 to 0.90 mg/kg, with most adverse events being of mild to moderate intensity [10]. Given its increased potency relative to propofol, ciprofol necessitates a lower drug volume for achieving anesthesia, which not only reduces the required solvent volume but may also mitigate side effects, particularly those associated with injection site pain.

The primary objective of this comprehensive meta-analysis is to systematically review and synthesize the existing body of literature pertaining to the safety and efficacy of ciprofol compared to propofol in the context of induction and maintenance of general anesthesia in adult patients undergoing surgical procedures. Through the amalgamation of data from multiple studies, we aspire to offer an extensive evaluation of the relative merits of these two agents. By doing so, we aim to provide valuable insights for both researchers and clinicians in the field of anesthesiology, ultimately contributing to the enhancement of anesthesia practices and patient care.

## Methods

### Data sources and search strategy

**Fig. 1:**
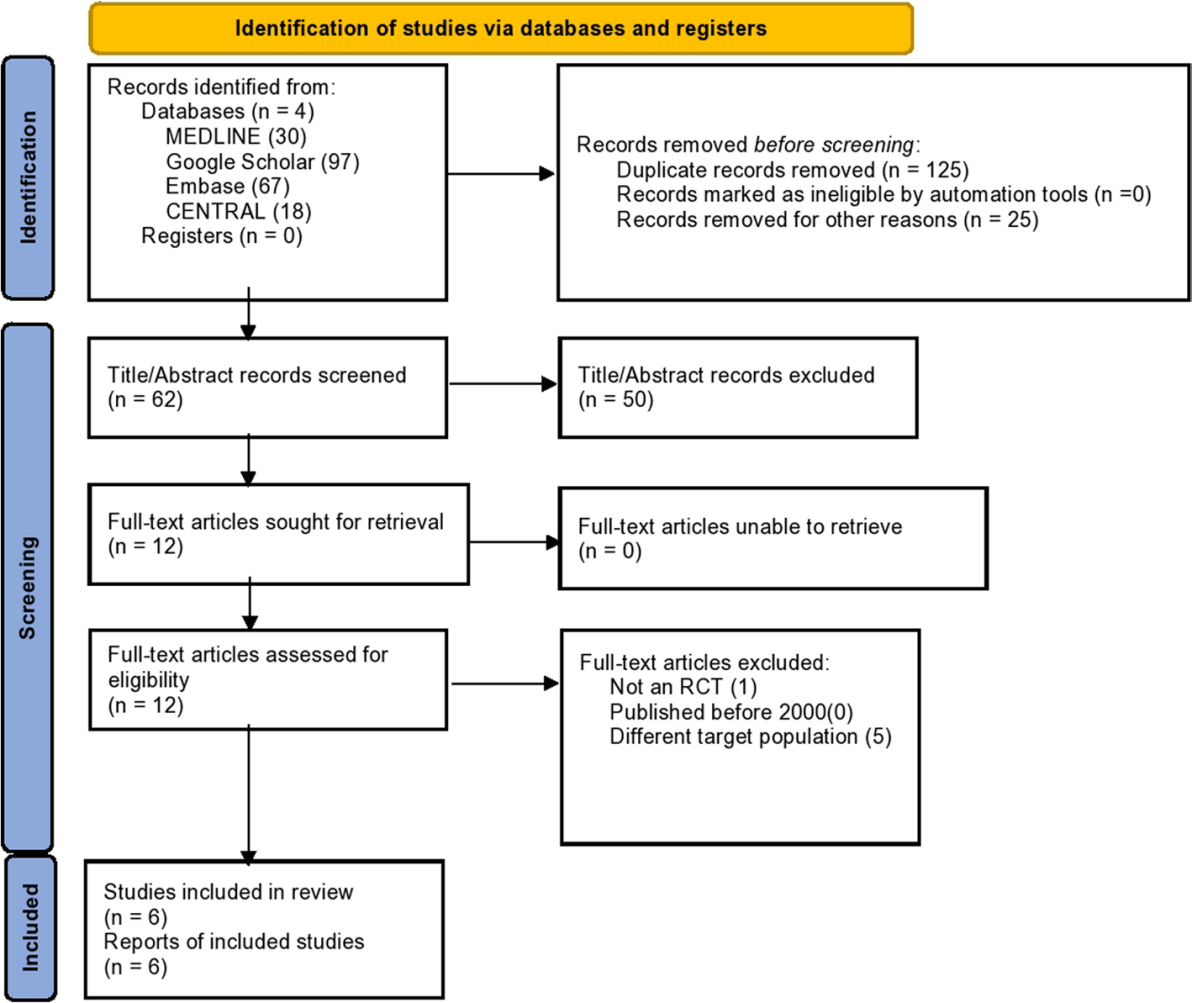
Prisma flow chart

Cochrane and Preferred Reporting Items for Systematic Review and Meta-Analyses (PRISMA) guidelines were implemented while preparing this meta-analysis [11]. A comprehensive electronic search performed using Medline, Google Scholar, Embase, and Cochrane Central was conducted to identify relevant randomized controlled trials (RCTs). The search strategy was composed of the following keywords and their MeSH terms “Propofol” OR “2,6-Diisopropylphenol” OR “2,6 Diisopropylphenol” OR “2,6-Bis(1-methyl ethyl)phenol” OR “Disoprofol” OR “Diprivan” OR “Disoprivan” OR “Fresofol” OR “ICI-35,868” OR “ICI 35,868” OR “ICI35,868” OR “ICI-35868” OR “ICI 35868” OR “ICI35868” OR “Ivofol” OR “Propofol Fresenius” OR “Propofol MCT” OR “Propofol Rovi” OR “Propofol-Lipuro” OR “Recofol” OR “Aquafol” OR “Propofol Abbott” AND “ciprofol OR HSK3486” AND “anesthesia OR sedation”. The PRISMA diagram of the studies used can be found in the PRISMA flow chart in Fig. 1. Information about the search strategy is given in Supplementary Table S1.

### Eligibility criteria

The study selection process was conducted in accordance with predetermined eligibility criteria and specific outcome measures. Only double-arm, randomized controlled trials (RCTs) were included in our analysis. The target demographic comprised individuals aged eighteen or over. The intervention involved the utilization of ciprofol, which was compared with the administration of propofol. The primary outcome assessed was the induction and maintenance of general anesthesia. Some studies were omitted based on the exclusion criteria. Studies in which ciprofol was utilized for screening and diagnostic procedures were not included. Articles published in languages other than English or any other specified language were excluded from consideration. Furthermore, all types of reviews (systematic and non-systematic), case reports, case series, cross-sectional, editorials, commentaries, and animal studies were excluded to maintain the integrity and focus of our study. Details of the studies that were selected are given in Supplementary Table S2. This rigorous selection process aimed to ensure the quality and relevance of the studies included in our systematic review and meta-analysis.

### Data extraction and quality assessment

Articles retrieved from the systematic search were exported to EndNote Reference Library software, and any duplicates found were discarded. The remaining articles were initially screened based on abstract and title, and then a review of the entire text was conducted to assess relevance. Screening of the articles was distributed amongst two reviewers, (M.H, H.M), and any inconsistencies were resolved by discussion till consensus or by the third reviewer (A.R.S.S). The following baseline characteristics were extracted onto an online Microsoft Excel Spreadsheet: study characteristics (first author’s name along with publication year, study design, number of patients) population characteristics (patient age in years, male gender percentage, body mass index (BMI) (kg/m^2^), American Society of Anesthesiologists (ASA) score, mean operation time, subgroups of dosage of drug). The baseline characteristics are given in Supplementary Table S3.

Primary outcomes included efficacy of ciprofol (satisfaction evaluation for anesthesiologists, time to full alertness, time to successful anesthesia induction, time to loss of eyelash reflex, success rate of anesthesia, time required for patients to leave the post-anesthesia care unit - PACU, time to respiratory recovery) on anesthesia induction and maintenance in comparison to propofol.

Secondary outcomes included the safety profile of ciprofol (total adverse events, tachycardia, rash, prolonged QT interval, pain on injection (induction), hypoxia, hypotension, hypertension (induction), Common Terminology Criteria for Adverse Events (CTCAE) severity scale (grade 1) (induction), CTCAE severity scale (grade 2) (induction), bradycardia (induction), any treatment-emergent adverse event, number of patients who maintained BIS between 40 and 60 min at 0.4 mg/kg and elevated AST (induction and maintenance) at 0.4 mg/kg. This is shown in Supplementary Table S4.

The revised Cochrane Risk of Bias (RoB) tool was used independently by the two researchers (H.M, N.F.S) to examine the quality of the included RCTs [12]. Reports were analyzed for the generation of allocation sequence, randomization of participants to ciprofol (intervention group) or propofol (control group), selective reporting of outcomes, and missing data.

### Statistical analysis

For the statistical analysis of the extracted data, we employed RevMan 5.4.1. In instances where raw data was available, we calculated risk ratios (RR) and mean difference (MD) along with their corresponding 95% confidence intervals (CIs). These calculations were performed using a random-effects model, allowing us to create forest plots that visually represented the dichotomous and continuous outcomes respectively.

Heterogeneity was measured using the Higgins *I*^2^ statistics and was reported as a percentage for every outcome. For an *I*^2^ value of less than 50%, low heterogeneity was indicated, moderate heterogeneity was considered when the *I*^2^ value was less than 75%, and high heterogeneity was observed with an *I*^2^ value greater than 75%. Outcomes, if reporting an *I*^2^ greater than 75%, were subjected to sensitivity analysis. Following the high heterogeneity leave one out sensitivity analysis was performed for only one outcome time to successful anesthesia induction.

In all statistical analyses, a *p* value of ≤ 0.05 was established as the threshold for statistical significance. This criterion was applied across the board to determine the significance of our findings.

### Publication bias

To assess for publication bias, we generated funnel plots for all outcomes using the random effects model. Funnel plots for the primary outcomes are available in the supplementary material (Supplementary Figures S2–S8).

## Results

### Eligible studies

In adherence to predetermined eligibility criteria and specific outcome measures, our meta-analysis considered six double-arm, RCTs [13–18]. These trials investigated the use of ciprofol versus propofol for the induction and maintenance of general anesthesia (Fig. 1).

### Baseline characteristics

Our thorough analysis encompassed the mentioned RCTs, involving a total of 714 participants. Among them, 316 patients received propofol 2 mg/kg, while 381 patients were administered ciprofol (HSK3486). Among the individuals in the intervention group, 64 patients were administered 0.5 mg/kg of ciprofol, while the remainder received 0.4 mg/kg. The average age of the study population was 39.5 years. However, it included patients of ages above 18. Gender distribution data revealed that approximately 65.4% of the participants were females. The majority of participants exhibited an ASA score of 2, indicating a good general health condition. A comprehensive summary of the baseline characteristics of the included patients can be found in Supplementary Table S3.

### Primary outcomes

#### Satisfaction evaluation for anesthesiologists

Analysis on satisfaction evaluation for anesthesiologists incorporated data from two studies, [13, 14]. On average, no significant difference was observed in terms of the anesthesiologist satisfaction levels when using ciprofol compared to propofol for anesthesia induction and maintenance (MD 0.14; 95% CI − 0.28 to 0.5; *p* = 0.51; *I*^2^ = 9%). Subgroup analysis unveiled a similar trend, with no significant difference in preference for either ciprofol or propofol during the induction phase (MD 0.40; 95% CI − 0.56 to 1.36; *p* = 0.42; *I*^2^ = 47%), or the maintenance phase (MD − 0.10, 95% CI − 1.00 to 0.80).

#### Satisfaction of the anesthesiologist (Fig. 2)

**Fig. 2:**
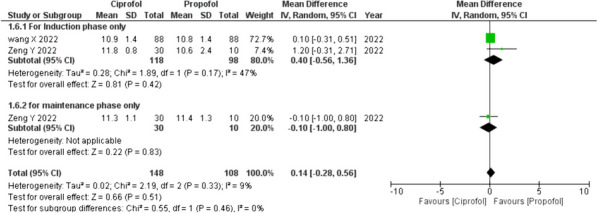
Satisfaction evaluation for anesthesiologists (forest plot)

Two studies reported these data [14, 15]. In both, there were no statistically significant differences in patients who received either drug with regards to achieving full alertness. The MD 0.11 min; 95% CI − 1.29 to 1.52; *p* = 0.87; *I*^2^ = 0%.

#### Time to successful anesthesia induction

The comprehensive analysis, drawing data from five out of six studies [13–17], highlighted a significant advantage of propofol. The time required for a successful anesthesia induction was significantly shorter with propofol compared to ciprofol, with a mean difference of 0.08 min (95% CI 0.00 to 0.15, *p* = 0.04, *I*^2^ = 77%). Subgroup analyses however showed that there was a statistically significant difference with ciprofol 0.5 mg/kg resulting in a shorter time to induction for propofol and no difference in the ciprofol 0.4 mg/kg group. Interestingly, a leave-one-out sensitivity analysis evidenced one study [13] as a source of substantial heterogeneity within the subgroup receiving ciprofol 0.4 mg/kg of treatment. Upon its removal, subgroup-specific heterogeneity significantly decreased to 0%, and overall heterogeneity had a minor reduction to 73%.

#### Time to loss of eyelash reflex (Fig. 3)

**Fig. 3:**
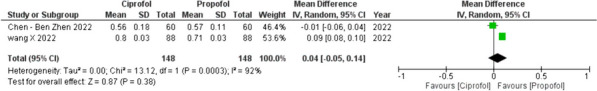
Time to loss of eyelash reflex (forest plot)

Analysis of data reported in two studies revealed that there was no difference observed between the two drugs in terms of time to loss of eyelash reflex (95% CI − 0.05 to 0.14; *p* = 0.38; *I*^2^ = 92%) [13, 16]. Unfortunately, due to the pronounced heterogeneity and limited data, a leave-one-out analysis was not feasible.

#### Success rate of anesthesia (Figs. 4 and 5)

**Fig. 4:**
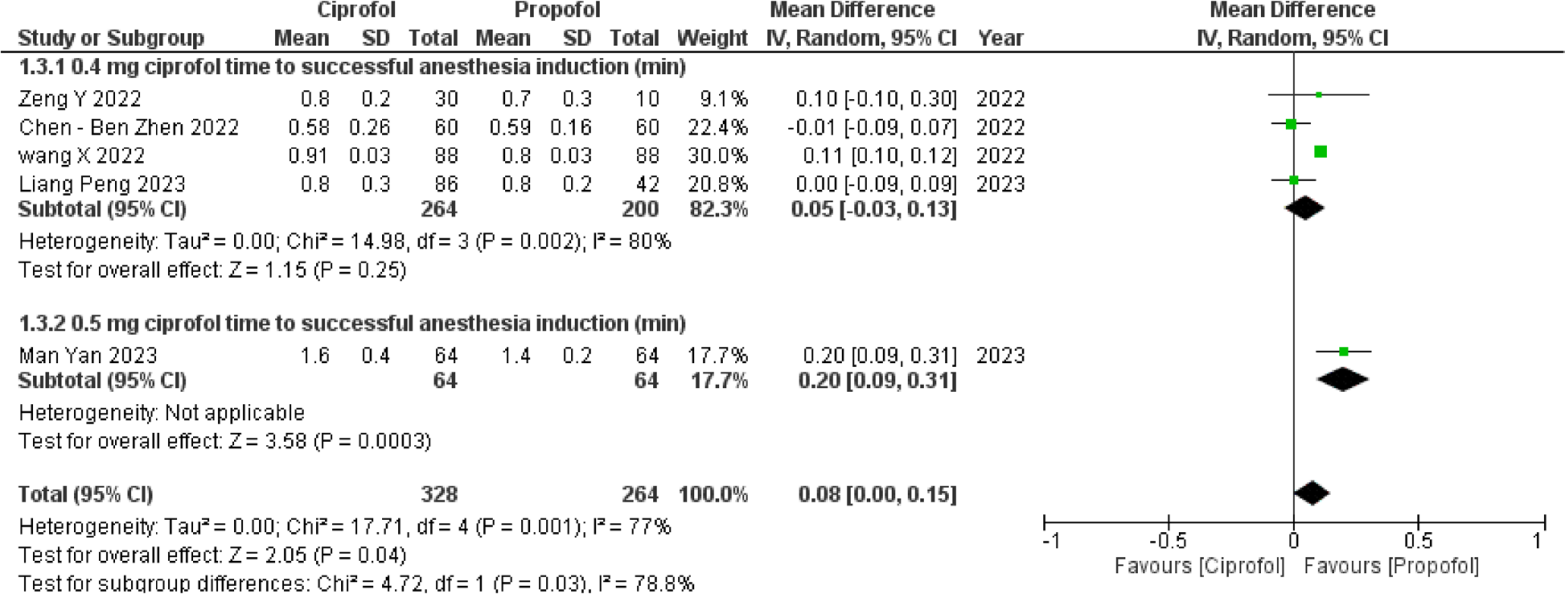
Time to successful anesthesia induction (forest plot)

**Fig. 5:**
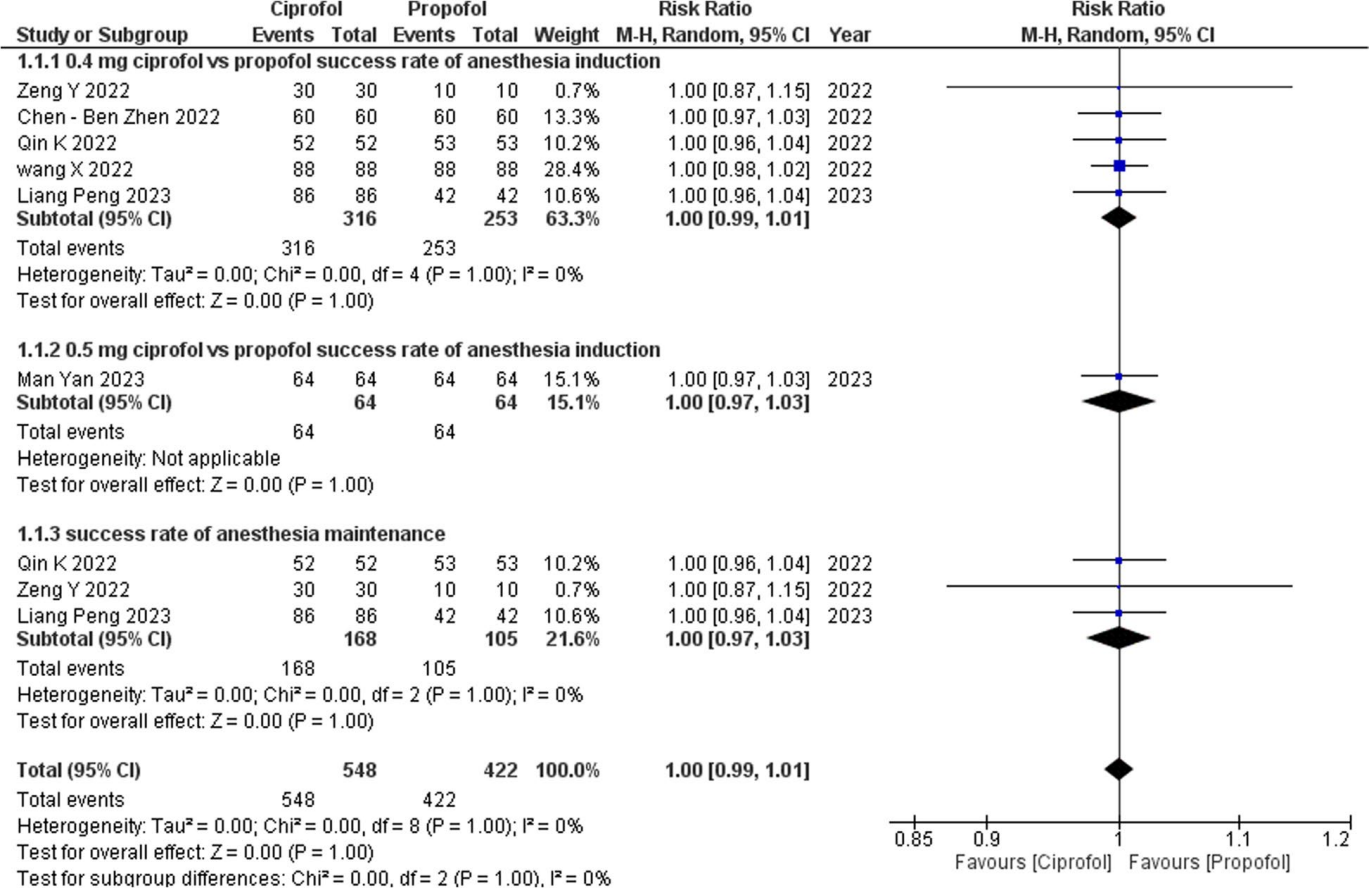
Success rate of anesthesia (forest plot)

Across all six studies [13–18], the combined analysis demonstrated no discernible differences between ciprofol and propofol in terms of the success rate of anesthesia induction and maintenance. The risk ratio (RR) was 1.00, with a 95% CI of 0.99 to 1.01 (*p* = 1.00, *I*^2^ = 0%). Subgroup analysis further reinforced these findings, revealing no significant differences at different dosage levels of ciprofol (0.4 mg/kg and 0.5 mg/kg) for both the induction and maintenance phases.

#### Time to full alertness (Fig. 6)

**Fig. 6:**
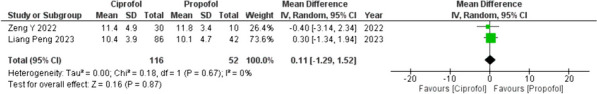
Time to full alertness (forest plot)

Based on data from two studies [14, 15], the pooled analysis indicated that there were no differences with regard to the time required to leave the PACU between patients who received propofol and those administered ciprofol at both dosages (95% CI − 1.45 to 2.34, *p* = 0.64, *I*^2^ = 0%).

#### Time to respiratory recovery (Fig. 7)

**Fig. 7:**
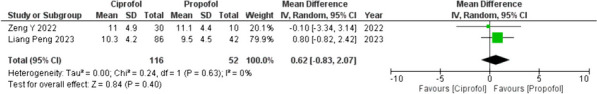
Time to respiratory recovery (forest plot)

**Fig. 8:**
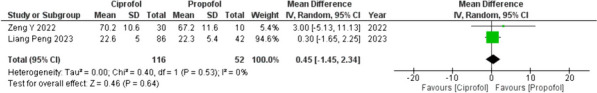
Time required for patients to leave the post-anesthesia care unit (PACU) (forest plot)

Analyzing data from two studies [14, 15] comparing ciprofol 0.4 mg/kg to propofol, there was no statistically significant difference in recovery time for respiratory functions following both induction and maintenance phases (*p* = 0.40). Time required for patients to leave the post-anesthesia care unit (PACU) (Fig. 8).

### Secondary outcomes

Upon performing the analysis, no significant differences were found in all of the outcomes except pain on the injection site in which ciprofol performed significantly better in having less pain (*P* = 0.0003). Insignificant differences between the two drugs were revealed in terms of total adverse events, tachycardia, rash, prolonged QT interval, hypoxia, hypotension, hypertension, CTCAE severity grading (grade 1), CTCAE severity grading (grade 2), bradycardia, any treatment-emergent adverse events, elevated aspartate transaminase (AST), number of patients who maintained bispectral index (BIS) between 40 and 60 min.

Furthermore, after conducting a subgroup analysis it was discovered that a significant reduction in total adverse events occurred when 0.5 mg/kg ciprofol was used for induction (*P* < 0.0001). Similarly, ciprofol was significantly better in terms of reducing the incidence of tachycardia when 0.5 mg/kg ciprofol was utilized for both induction and maintenance of general anesthesia (*P* = 0.01). Propofol performed significantly worse compared to 0.4 mg/kg ciprofol during the induction phase according to the CTCAE severity grading scale (grade 1) (*P* = 0.005). The results are mentioned in Supplementary Table S4.

### Quality assessment

We conducted a rigorous quality assessment of the included trials using the Cochrane risk of bias tool, identifying trials of moderate-to-high quality. The Cochrane risk of bias tool assessed the included randomized controlled trials (RCTs), indicating a low risk of bias in selection, performance, and reporting domains.

### Publication bias

Funnel plots were made to evaluate the presence of publication bias. The funnel plots visually displayed the distribution of studies for each outcome, with the vertical axis representing the effect size and the horizontal axis representing the precision of the estimates. In general, the funnel plots exhibited an equal and symmetrical distribution of studies on both sides of the vertical axis, indicating no significant publication bias for all outcomes (Supplementary Figures S2–S8).

## Discussion

The influence of anesthesiologists' satisfaction is pivotal in selecting anesthetic agents. Their trust in a drug's effectiveness and safety profoundly impacts patient care. Our meta-analysis hints at a slightly stronger preference for ciprofol, particularly during the induction phase. It is essential to note, though, that these preferences don't quite reach the threshold of statistical significance thus emphasizing that ciprofol and propofol exhibit similar satisfaction levels among anesthesiologists during both induction and maintenance phases of anesthesia. This aligns with existing literature, suggesting that ciprofol could be a compelling alternative to propofol in clinical anesthesia [19]. These consistent results strengthen the evidence that ciprofol can be a viable alternative to propofol in anesthesia practice, offering similar satisfaction levels for anesthesiologists while providing potential benefits such as safety and effectiveness [20, 21]. However, the absence of statistical significance highlights the multifaceted nature of this preference. Various factors, including individual preferences, patient-specific characteristics, surgical requirements, and the collective experiences of the anesthesia team, all play a role in shaping satisfaction levels. Furthermore, variations in satisfaction at different phases of anesthesia administration emphasize the need for tailored approaches to match the unique demands of each surgical step, ensuring the best possible patient outcomes and overall satisfaction [22].

Interestingly, in this study, no statistically significant difference between both drugs for alertness were detected. However, these results are inconsistent with other existing literature on the subject. For instance, a recent systematic review [19] in the context of painless gastroenteroscopy found that propofol consistently leads to faster alertness compared to ciprofol. This inconsistency in results could be attributed to the fact that our study exclusively focused on invasive surgeries, which encompassed a diversity of surgical types. Propofol is well-known for its characteristics of rapid onset and swift recovery, which results from its pharmacokinetic property of fast elimination [3, 23]. Thus, it is an important option to induce and maintain anesthesia, particularly for short-duration procedures. The rapid elimination of propofol minimizes the risk of residual sedation, making it a valuable drug also for cesarean delivery [24]. Anesthesiologists value propofol for its ability to induce and reverse anesthesia swiftly, providing a significant advantage in various clinical scenarios. However, it's crucial to recognize that this advantage comes with the caveat of a relatively narrow therapeutic window and potential concentration-dependent effects on cardiovascular and respiratory systems, especially in elderly and frail patients [9]. These considerations underscore the importance of a nuanced approach when selecting anesthetic agents, taking into account the specific characteristics and vulnerabilities of the patient population.

Propofol's superior induction speed, consistent with previous research, highlights its status as the preferred choice for anesthesia induction in clinical practice [19]. The absence of a substantial difference in induction time between ciprofol at 0.4 mg/kg and propofol is an intriguing finding. It suggests that ciprofol can achieve induction times similar to propofol at 0.4 mg/kg [15]. Heterogeneity is observed in a study for several reasons [13]. Firstly, the researchers conducted a phase 3, multicenter, randomized, double-blind, comparative study, which introduced differences in study design, data collection, and interpretation compared to studies in the same analysis. Additionally, the study had a larger sample size with a higher percentage of male patients, potentially introducing gender-related variations in anesthesia induction times, due to differences in drug responses and pharmacokinetics. Hormonal fluctuations, such as those associated with the menstrual cycle in females, can impact drug metabolism and distribution. This can result in variations in anesthesia induction times between male and female patients [25]. Furthermore, variations in patient age, BMI, and ASA score distribution in that study could impact how individuals respond to anesthesia, leading to differences in induction times.

Contrary to the above-mentioned findings, our focus on the loss of eyelash reflex specifically revealed no divergences between the two agents. However, it is essential to acknowledge the presence of pronounced heterogeneity in the analysis of this parameter, which suggests substantial variability among the included studies in this meta-analysis. This heterogeneity, coupled with the limitation of limited data availability resulted in a trend not favoring either of the drugs, especially propofol.

This meta-analysis provides valuable insights, affirming that both ciprofol and propofol can effectively serve for anesthesia induction and maintenance, with no significant differences observed. This conclusion gains strength through the subgroup analysis, which demonstrates that even with different ciprofol dosages (0.4 mg/kg or 0.5 mg/kg), there are no significant differences in the success rate of anesthesia induction compared to propofol. This suggests that the choice of ciprofol dosage doesn't significantly affect induction success rates [26]. These findings hold practical implications for anesthesiologists, indicating that both ciprofol and propofol are valid choices for anesthesia induction and maintenance. Clinicians can make their choices based on patient-specific factors and individual preferences.

The observation of a faster exit from the PACU and improved recovery of respiratory functions with propofol aligns with its established characteristics of rapid onset and short duration of action. This can be attributed to propofol's favorable pharmacokinetic profile. However, the absence of statistical significance in these findings could be due to inherent variability in patient responses and the specific criteria used for assessment [27]. Nevertheless, these findings have significant clinical relevance, as quicker recovery and discharge from the PACU can enhance patient throughput and optimize resource utilization [28].

Pain at the injection site, a factor that can induce anxiety and discomfort among patients during intravenous infusion, is a critical consideration. Propofol has been known to cause pain at the injection site [29]. To mitigate this side effect, injection of local anesthetics such as lidocaine before intravenous propofol administration, as well as the use of a more diluted propofol, have been considered for pain reduction at the injection site [30]. In support of the current literature, this meta-analysis collectively shows that ciprofol is less likely to cause pain at the injection site. This can be explained by its hydrophobic nature, resulting in relatively lower plasma concentrations compared to propofol [31].

In this comprehensive meta-analysis comparing ciprofol and propofol for anesthesia, the safety profiles of these two drugs were assessed with a thorough evaluation of various adverse events. The findings indicate that, in general, there was no statistically significant difference observed between ciprofol and propofol in terms of overall adverse events. This suggests that both agents are generally well-tolerated and safe for use in anesthesia induction. Comparing our results to the existing literature, studies have reported varying safety profiles for both ciprofol and propofol. Some have highlighted the safety and effectiveness of ciprofol in anesthesia induction, with a lower incidence of adverse events [26]. In contrast, others have noted that propofol remains a standard and safe choice for anesthesia induction [32].

This study has some limitations. In order to increase the quality of the extracted data, we only included double-arm randomized control trials, limiting our dataset to six studies. Additionally, it exclusively focused on invasive surgeries, which encompassed a diversity of surgical types. Since some studies focused on induction of anesthesia while others studied both induction and maintenance of sedation through ciprofol, our choice of primary safety outcomes was limited. Though most studies affirm the safety of both drugs for clinical practice, it is worth noting that the existing literature on the comparison between these two drugs is relatively limited. Looking ahead, future research should delve into optimized ciprofol dosing strategies aimed at achieving the desired depth of anesthesia while minimizing side effects [33]. Exploring patient-centered outcomes and integrating advanced monitoring technologies could also provide deeper insights into the comparative strengths and weaknesses of these agents. Large-scale studies spanning diverse patient groups and clinical scenarios, including specific procedures like sedation for gastrointestinal endoscopy or other procedures, can shed light on the advantages concerning patient comfort and recovery [34]. Moreover, investigating long-term outcomes and cost-effectiveness can offer valuable guidance for clinical decision-making.

## Conclusion

In conclusion, this systematic review and meta-analysis have highlighted the comparative effectiveness and safety of ciprofol and propofol in the context of general anesthesia. Propofol had a faster onset of anesthesia during the induction phase. Conversely, ciprofol resulted in a reduced incidence of pain at the injection site. Clinicians should consider these findings while tailoring their choice of anesthetic agents to individual patient characteristics and preferences.

### Supplementary Information

**Additional file 1:** **Supplementary Figure S1.** Cochrane Risk of Bias. **Supplementary Figure S2**. Satisfaction Evaluation for Anesthesiologists (funnel plot). **Supplementary Figure S3** Time to Full Alertness (funnel plot). **Supplementary Figure S4.** Time to Successful Anesthesia Induction (funnel plot). **Supplementary Figure S5.** Time to loss of Eyelash Reflex (funnel plot). **Supplementary Figure S6.** Success Rate of Anesthesia (funnel plot). **Supplementary Figure S7.** Time Required for Patients to Leave the Post-Anesthesia Care Unit (PACU) (funnel plot). **Supplementary Figure S8.** Time to Respiratory recovery (funnel plot). **Supplementary Table S1.** Search strategy. **Supplementary Table S2.** Details of the studies. **Supplementary Table S3.** Baseline characteristics. **Supplementary Table S4**. Study outcomes.

## References

[1] Grasshoff C, Rudolph U, Antkowiak B (2005). Molecular and systemic mechanisms of general anaesthesia: the 'multi-site and multiple mechanisms' concept. Curr Opin Anaesthesiol.

[2] Mashour GA, Forman SA, Campagna JA (2005). Mechanisms of general anesthesia: from molecules to mind. Best Pract Res Clin Anaesthesiol.

[3] Sahinovic MM, Struys MMRF, Absalom AR (2018). Clinical pharmacokinetics and pharmacodynamics of propofol. Clin Pharmacokinet.

[4] Baker MT, Naguib M (2005). Propofol: the challenges of formulation. Anesthesiology.

[5] Doenicke AW, Roizen MF, Rau J, Kellermann W, Babl J (1996). Reducing pain during propofol injection: the role of the solvent. Anesth Analg.

[6] Robinson BJ, Ebert TJ, Obrien TJ, Colinco MD, Muzi M (1997). Mechanisms whereby propofol mediates peripheral vasodilation in humans. Sympathoinhibition or direct vascular relaxation?. Anesthesiology.

[7] Diedrich DA, Brown DR (2011). Analytic reviews: propofol infusion syndrome in the ICU. J Intensive Care Med.

[8] Qin L, Ren L, Wan S, Liu G, Luo X, Liu Z, Li F, Yu Y, Liu J, Wei Y (2017). Design, synthesis, and evaluation of novel 2,6-disubstituted phenol derivatives as general anesthetics. J Med Chem.

[9] Teng Y, Ou M, Wang X, Zhang W, Liu X, Liang Y, Li K, Wang Y, Ouyang W, Weng H, Li J, Yao S, Meng J, Shangguan W, Zuo Y, Zhu T, Liu B, Liu J (2021). Efficacy and safety of ciprofol for the sedation/anesthesia in patients undergoing colonoscopy: phase IIa and IIb multi-center clinical trials. Eur J Pharm Sci.

[10] Teng Y, Ou MC, Wang X (2021). Pharmacokinetic and pharmacodynamic properties of ciprofol emulsion in Chinese subjects: a single center, open-label, single-arm dose-escalation phase 1 study. Am J Transl Res.

[11] Liberati A, Altman DG, Tetzlaff J, Mulrow C, Gøtzsche PC, Ioannidis JPA, Clarke M, Devereaux PJ, Kleijnen J, Moher D (2009). The PRISMA statement for reporting systematic reviews and meta-analyses of studies that evaluate healthcare interventions: explanation and elaboration. BMJ.

[12] Sterne JAC, Savović J, Page MJ, Elbers RG, Blencowe NS, Boutron I, Cates CJ, Cheng HY, Corbett MS, Eldridge SM, Emberson JR, Hernán MA, Hopewell S, Hróbjartsson A, Junqueira DR, Jüni P, Kirkham JJ, Lasserson T, Li T, McAleenan A, Reeves BC, Shepperd S, Shrier I, Stewart LA, Tilling K, White IR, Whiting PF, Higgins JPT (2019). RoB 2: a revised tool for assessing risk of bias in randomised trials. BMJ.

[13] Wang X, Wang X, Liu J, Zuo YX, Zhu QM, Wei XC, Zou XH, Luo AL, Zhang FX, Li YL, Zheng H, Li H, Wang S, Wang DX, Guo QL, Liu CM, Wang YT, Zhu ZQ, Wang GY, Ai YQ, Xu MJ (2022). Effects of ciprofol for the induction of general anesthesia in patients scheduled for elective surgery compared to propofol: a phase 3, multicenter, randomized, double-blind, comparative study. Eur Rev Med Pharmacol Sci.

[14] Zeng Y, Wang DX, Lin ZM, Liu J, Wei XC, Deng J, Liu YF, Ma EL, Yang MC, Zheng H, Yu XD, Guo QL, Guan YJ (2022). Efficacy and safety of HSK3486 for the induction and maintenance of general anesthesia in elective surgical patients: a multicenter, randomized, open-label, propofol-controlled phase 2 clinical trial. Eur Rev Med Pharmacol Sci.

[15] Liang P, Dai M, Wang X, Wang D, Yang M, Lin X, Zou X, Jiang K, Li Y, Wang L, Shangguan W, Ren J, He H (2023). Efficacy and safety of ciprofol vs. propofol for the induction and maintenance of general anaesthesia: a multicentre, single-blind, randomised, parallel-group, phase 3 clinical trial. Eur J Anaesthesiol.

[16] Chen BZ, Yin XY, Jiang LH, Liu JH, Shi YY, Yuan BY (2022). The efficacy and safety of ciprofol use for the induction of general anesthesia in patients undergoing gynecological surgery: a prospective randomized controlled study. BMC Anesthesiol.

[17] Man Y, Xiao H, Zhu T, Ji F (2023). Study on the effectiveness and safety of ciprofol in anesthesia in gynecological day surgery: a randomized double-blind controlled study. BMC Anesthesiol.

[18] Qin K, Qin WY, Ming SP, Ma XF, Du XK (2022). Effect of ciprofol on induction and maintenance of general anesthesia in patients undergoing kidney transplantation. Eur Rev Med Pharmacol Sci.

[19] Chen X, Guo P, Yang L, Liu Z, Yu D (2022). Comparison and clinical value of ciprofol and propofol in intraoperative adverse reactions, operation, resuscitation, and satisfaction of patients under painless gastroenteroscopy anesthesia. Contrast Media Mol Imaging.

[20] Hu C, Ou X, Teng Y, Shu S, Wang Y, Zhu X, Kang Y, Miao J (2021). Sedation effects produced by a ciprofol initial infusion or bolus dose followed by continuous maintenance infusion in healthy subjects: a phase 1 trial. Adv Ther.

[21] Lu M, Liu J, Wu X, Zhang Z (2023). Ciprofol: a novel alternative to propofol in clinical intravenous anesthesia?. Biomed Res Int.

[22] Lemos JN, Lemos LDCN, Solla DJF, Lemos DDCN, Módolo NSP (2023). Patient satisfaction in ambulatory anesthesia assessed by the Heidelberg Peri-anaesthetic Questionnaire: a cross-sectional study. Braz J Anesthesiol.

[23] Fulton B, Sorkin EM (1995). Propofol. An overview of its pharmacology and a review of its clinical efficacy in intensive care sedation. Drugs.

[24] Celleno D, Capogna G, Emanuelli M, Varrassi G, Muratori F, Costantino P, Sebastiani M (1993). Which induction drug for cesarean section? A comparison of thiopental sodium, propofol, and midazolam. J Clin Anesth.

[25] Mittelstrass K, Ried J, Yu Z, Krumsiek J, Gieger C, Prehn C, Roemisch-Margl W, Polonikov A, Peters A, Theis F, Meitinger T, Kronenberg F, Weidinger S, Wichmann H, Suhre K, Wang-Sattler R, Adamski J, Illig T (2011) Discovery of sexual dimorphisms in metabolic and genetic biomarkers. PLoS Genet 7. 10.1371/journal.pgen.1002215 10.1371/journal.pgen.1002215 PMC3154959 21852955

[26] Li J, Wang X, Liu J, Wang X, Li X, Wang Y, Ouyang W, Li J, Yao S, Zhu Z, Guo Q, Yu Y, Meng J, Zuo Y (2022). Comparison of ciprofol (HSK3486) versus propofol for the induction of deep sedation during gastroscopy and colonoscopy procedures: a multi-centre, non-inferiority, randomized, controlled phase 3 clinical trial. Basic Clin Pharmacol Toxicol.

[27] Karcz M, Papadakos PJ (2013). Respiratory complications in the postanesthesia care unit: a review of pathophysiological mechanisms. Can J Respir Ther.

[28] Zhou Q, Han Y, Chen J (2022). Meta-analysis of anesthetic efficacy and safety of propofol in craniotomy patients. Contrast Media Mol Imaging.

[29] Zhu Q, Luo Z, Wang X, Wang D, Li J, Wei X, Tang J, Yao S, Ouyang W, Zhang W, Zuo Y, Wang X, Liu J (2023). Efficacy and safety of ciprofol versus propofol for the induction of anesthesia in adult patients: a multicenter phase 2a clinical trial. Int J Clin Pharm.

[30] Jalota L, Kalira V, George E (2011). Prevention of pain on injection of propofol: systematic review and meta-analysis. BMJ.

[31] Chen L, Xie Y, Du X, Qin W, Huang L, Dai J (2023). The effect of different doses of ciprofol in patients with painless gastrointestinal endoscopy. DDDT.

[32] Liu GL, Wu GZ, Ge D, Zhou HJ, Cui S, Gao K, Sun WJ, Yu DH, Liu SB, Liu JJ (2023). Efficacy and safety of ciprofol for agitation and delirium in the ICU: a multicenter, single-blind, 3-arm parallel randomized controlled trial study protocol. Front Med (Lausanne).

[33] Duan G, Lan H, Shan W, Wu Y, Xu Q, Dong X, Mei P, You M, Jin L, Wu J (2023). Clinical effect of different doses of ciprofol for induction of general anesthesia in elderly patients: a randomized, controlled trial. Pharmacol Res Perspect.

[34] Qin X, Lu X, Tang L, Wang C, Xue J (2023). Ciprofol versus propofol for sedation in gastrointestinal endoscopy: protocol for a systematic review and meta-analysis. BMJ Open.

