# Correction: Efficacy and safety of ciprofol versus propofol for induction and maintenance of general anesthesia: a systematic review and meta-analysis

**DOI:** 10.1186/s44158-024-00162-6

**Published:** 2024-04-24

**Authors:** Muhammad Hudaib, Hurais Malik, Syeda Javeria Zakir, Samra Rabbani, Dhanushan Gnanendran, Abdul Rehman Shah Syed, Noor Fatima Suri, Javeria Khan, Arham Iqbal, Nowal Hussain, Muhammad Abdullah, Satesh Kumar, Mahima Khatri, Giustino Varrassi

**Affiliations:** 1 Fazaia Ruth Pfau Medical College, Karachi, Pakistan; 2 Dow University of Health Sciences, Karachi, Pakistan; 3 York and Scarborough Teaching Hospital, NHS Foundation Trust, York, UK; 4 CMH Lahore Medical College and Institute of Dentistry, Lahore, Pakistan; 5 Shaheed Mohtarma Benazir Bhutto Medical College, Karachi, Pakistan; 6 Paolo Procacci Foundation (PPF), Rome, Italy

**Correction** **:** **J Anesth Analg Crit Care 4, 25 (2024)**

**https://doi.org/10.1186/s44158-024-00160-8**

In the original publication of this article [1] the author names were inversed. The incorrect and correct names are shown in this article, the original article has been updated.

Incorrect

- Hudaib Muhammad, Malik Hurais, Syeda Javeria Zakir, Rabbani Samra, Gnanendran Dhanushan, Syed Abdul Rehman Shah, Noor Fatima Suri, Khan Javeria, Iqbal Arham, Hussain Nowal, Abdullah Muhammad, Kumar Satesh, Khatri Mahima & Varrassi Giustino

Correct

- Muhammad Hudaib, Hurais Malik, Syeda Javeria Zakir, Samra Rabbani, Dhanushan Gnanendran, Abdul Rehman Shah Syed, Fatima Noor Suri, Javeria Khan, Arham Iqbal, Nowal Hussain, Muhammad Abdullah, Satesh Kumar, Mahima Khatri & Giustino Varrassi
