# Supplementary material for: Efficacy and safety of ciprofol versus propofol for induction and maintenance of general anesthesia: a systematic review and meta-analysis
Source: J Anesth Analg Crit Care. 2024 Apr 11;4:25. doi: 10.1186/s44158-024-00160-8 (PMC11008023; doi:10.1186/s44158-024-00160-8)
Supplement: Supplementary file 1 — Additional file 1: Supplementary Figure S1. Cochrane Risk of Bias. Supplementary Figure S2. Satisfaction Evaluation for Anesthesiologists (funnel plot). Supplementary Figure S3 Time to Full Alertness (funnel plot). Supplementary Figure S4. Time to Successful Anesthesia Induction (funnel plot). Supplementary Figure S5. Time to loss of Eyelash Reflex (funnel plot). Supplementary Figure S6. Success Rate of Anesthesia (funnel plot). Supplementary Figure S7. Time Required for Patients to Leave the Post-Anesthesia Care Unit (PACU) (funnel plot). Supplementary Figure S8. Time to Respiratory recovery (funnel plot). Supplementary Table S1. Search strategy. Supplementary Table S2. Details of the studies. Supplementary Table S3. Baseline characteristics. Supplementary Table S4. Study outcomes. [file 44158_2024_160_MOESM1_ESM.docx]

**Supplementary Table 1**

| **DATABASE** | **SEARCH STRATEGY** | **RESULTS** |
| --- | --- | --- |
| Medline | (((((((((((((((((((((Propofol) OR (2,6-Diisopropylphenol)) OR (2,6 Diisopropylphenol)) OR (2,6-Bis(1-methylethyl)phenol)) OR (Disoprofol)) OR (Diprivan)) OR (Disoprivan)) OR (Fresofol)) OR (ICI-35,868)) OR (ICI 35,868)) OR (ICI35,868)) OR (ICI-35868)) OR (ICI 35868)) OR (ICI35868)) OR (Ivofol)) OR (Propofol Fresenius)) OR (Propofol MCT)) OR (Propofol Rovi)) OR (Propofol-Lipuro)) OR (Recofol)) OR (Aquafol)) OR (Propofol Abbott) AND ((ciprofol OR HSK3486)) AND ((anesthesia OR sedation)) | 30 |
| Google Scholar | (((((((((((((((((((((Propofol) OR (2,6-Diisopropylphenol)) OR (2,6 Diisopropylphenol)) OR (2,6-Bis(1-methylethyl)phenol)) OR (Disoprofol)) OR (Diprivan)) OR (Disoprivan)) OR (Fresofol)) OR (ICI-35,868)) OR (ICI 35,868)) OR (ICI35,868)) OR (ICI-35868)) OR (ICI 35868)) OR (ICI35868)) OR (Ivofol)) OR (Propofol Fresenius)) OR (Propofol MCT)) OR (Propofol Rovi)) OR (Propofol-Lipuro)) OR (Recofol)) OR (Aquafol)) OR (Propofol Abbott) AND ((ciprofol OR HSK3486)) AND ((anesthesia OR sedation)) | 97 |
| Embase | (((((((((((((((((((((Propofol) OR (2,6-Diisopropylphenol)) OR (2,6 Diisopropylphenol)) OR (2,6-Bis(1-methylethyl)phenol)) OR (Disoprofol)) OR (Diprivan)) OR (Disoprivan)) OR (Fresofol)) OR (ICI-35,868)) OR (ICI 35,868)) OR (ICI35,868)) OR (ICI-35868)) OR (ICI 35868)) OR (ICI35868)) OR (Ivofol)) OR (Propofol Fresenius)) OR (Propofol MCT)) OR (Propofol Rovi)) OR (Propofol-Lipuro)) OR (Recofol)) OR (Aquafol)) OR (Propofol Abbott) AND ((ciprofol OR HSK3486)) AND ((anesthesia OR sedation)) | 67 |

**Supplementary Table 2**

| **Author name** | **ZENG** | **LIANG** | **MAN** | **CHEN** | **QIN** | **Wang** |
| --- | --- | --- | --- | --- | --- | --- |
| **Trial name** | Efficacy and safety of HSK3486 for the induction and maintenance of general anesthesia in elective surgical patients: a multicenter, randomized, open-label, propofol-controlled phase 2 clinical trial | Efficacy and safety of ciprofol vs. propofol for the induction and maintenance of general anesthesia  A multicenter, single-blind, randomized, parallel-group, phase 3 clinical trial | Study on the effectiveness and safety of ciprofol in anesthesia in gynecological day surgery: a randomized double-blind controlled study | The efficacy and safety of ciprofol use for the induction of general anesthesia in patients undergoing gynecological surgery: a prospective randomized controlled study | Effect of ciprofol on induction and maintenance of general anesthesia in patients undergoing kidney transplantation | Effects of ciprofol for the induction of general anesthesia in patients scheduled for elective surgery compared to propofol: a phase 3, multicenter, randomized, double-blind, comparative study |
| **Patient no.** | 40 | 129 | 128 | 120 | 120 | 176 |
| **Year of publication** | 2022 | 2023 | 2023 | 2022 | 2022 | 2022 |
| **Trial type** | a multicenter, randomized, open-label, propofol-controlled phase 2 clinical trial | A multicenter, single-blind, randomized, parallel-group, phase 3 clinical trial | a randomized double-blind controlled study | a prospective randomized controlled study | prospective, randomized, single-blind study | A multi-center, randomized, propofol-controlled, double-blind trial |
| **Trial number** | NCT04048811 | [NCT04511728](https://clinicaltrials.gov/ct2/show/NCT04511728). | ChiCTR2100053444 | ChiCTR2100045211 | ChiCTR2200058826 | NCT03808844 |
| **Inclusion criteria** | Age 18 -65 years old.  An American Society of Anesthesiologists (ASA) rating of Class I-III, endotracheally intubated under GA, and a blood loss of ≤ 1,000 mL were included. | (American Society of Anesthesiologists (ASA) classes I to II); age ≥18 years and ≤65 years; | Age (18 ~ 64years), with American Society of Anesthesiologists physical classification status I or II, BMI 18 to 28 kg/m2. | Adult females between the ages of 18 to 60 (ASA physical status: I or II) who were scheduled to undergo elective gynecological surgery under GA. | Patients who had a kidney transplant under GA with tracheal intubation. Age 18-65 years, (BMI) of 18-30 kg/m2 American Society of Anesthesiology (ASA) physical status of III-IV. | Age 18-64 years  (BMI) between 18 and 30 kg/m2, American Society of Anesthesiologists physical status of I or II  scheduled to undergo elective surgery under HA |
| **Exclusion criteria** | Scheduled to receive emergency, surgical procedures with contraindications to GA or with a history of previous anesthesia incidents were excluded. | Contraindications to GA; a history of anesthesia incidents; a disease history of diseases or issues in any systems that could increase the risk of sedation/anesthesia. | Patients were excluded if they suffered from propofol allergies, had significant diseases in various systems Women who were pregnant, or planning to become pregnant were excluded. | morbid obesity, egg/soy allergies, had significant diseases in various systems. Women who were pregnant, lactating, or planning to become pregnant within 1 month after the trial were excluded. | patients with liver, mental, nervous system diseases, coagulation dysfunctions, heart failure, respiratory failure, long-term use of sedatives or antidepressants, pregnant or lactating women, and unable to communicate or cooperate. | a history of allergy or hypersensitivity. Those who had clinically significant systemic diseases; pregnant or had a pregnancy plan within 1 month postoperatively; a family history of malignant hyperthermia; those who had surgery under GA within 4 weeks perioperatively;  who had previously received sedative/narcotic agents within 3 days of screening and had alcohol or drug abuse within 3 months perioperatively;  who had previously received drugs that could have affected the QT interval or induced/inhibited P450 or CYP2B6 within 2 weeks perioperatively. |
| **Treatment** | Randomly assigned to HSK3486 or propofol dosage groups in a ratio of 3:1. Drugs were administered as a bolus injection of 0.4 mg/kg (HSK3486) or 2.0 mg/ kg (propofol) for induction, followed by maintenance infusion. | Patients were given midazolam 0.04 mg kg−1 and sufentanil 0.3 μg kg−1 as preanesthetic medication. Anesthesia induction was then initiated with either HSK3486 or propofol. After successful induction, the muscle relaxant rocuronium bromide was administered at 0.6 mg kg−1, and endotracheal intubation was performed. HSK3486 or propofol was given at appropriate doses to maintain anesthesia. Remifentanil was administered at 0.1–0.3 μg/kg−1 min−1 for analgesia, and sufentanil and a muscle relaxant were added. bromide. | During anesthesia induction, the ciprofol group was infused at a time limit of 0.5 mg/kg for one minute, and the propofol group was infused at a time limit of 2 mg/kg for 1 min. | Intravenous midazolam (0.03 mg/kg) and sufentanil (0.3 μg/kg) were used to start general anesthesia induction, followed 2 min later by the manual injection of ciprofol (0.4 mg/kg) or medium-and long-chain triglyceride (MCT/LCT) propofol (2 mg/kg). Patients started receiving preoxygenation after intravenous midazolam and sufentanil being administrated. When spontaneous breathing disappeared, it switched to manual controlled breathing. i | The patients were randomized into a ciprofol group (group C) and a propofol group (group P). Anesthesia induction: group C had injected IV with ciprofol 0.4 mg/kg, group P had injected IV with propofol 2.0 mg/kg, while both groups had injected IV with sufentanil 0.4-0.5 μg/kg and cisatracurium 0.2 mg/kg. Anesthesia maintenance: ciprofol was injected IV with 0.8-2.4 mg•kg-1•h-1 in group C, propofol was injected IV with 4-12 mg•kg-1•h-1 in group P, while remifentanil was injected IV with 8-15 μg•kg-1 •h-1 and cisatracurium was injected IV with 0.1-0.2mg•kg-1•h-1, with the bispectral index | Optimal injected doses of 0.4 mg/kg of ciprofol and 2.0 mg/kg of propofol |
| **Primary efficacy outcome** | The success rate of anesthesia maintenance. | Noninferiority between the drugs was evaluated as the lower limit of the 95% confidence interval (CI) for the group difference. | (1) bradycardia (HR < 50 beats/min, > 30s); (2) Tachycardia (HR > 100 beats/min, > 30s) (3) Hypotension (30% reduction in SBP compared to baseline value); (4) Hypertension (SBP is 20% higher than baseline value); (5) injection pain at the site. (6) Intraoperative body movements | Safety and efficacy of ciprofol. Evaluation The success rate of general anesthesia induction was the primary outcome for the present study. | The success rate of sedation. | The anesthesia induction success. |
| **Secondary Efficacy outcome** | Times from discontinuation of HSK3486 or propofol maintenance to full alertness, respiratory recovery, extubation and reaching the goal of the Aldrete score. | Successful anesthetic induction, full alertness and spontaneous breathing recovery, time until leaving the postanesthetic care. | (1) success rate of induction of anesthesia, (2) the time of loss of consciousness (time of initiation of study drug infusion to MOAA/S ≤ 1), (3) time of awakening (time of drug discontinuation to extubation), (4) study drug top-up doses, (5) rescue drug use. | (1) the time to onset of successful induction; (2) the incidence of injection site pain as detected by a withdrawal response or a numeric rating. (3) time to eyelash reflex disappearance. (4) changes in the bispectral index (BIS) during the 10-min interval. | . HR showed no significant difference between the two groups (p>0.05). MAP decreased more significantly in group P at T6 (p0.05) | The average time to successful anesthesia and loss of the eyelash reflex.  The pattern of BIS changes.  The incidence of injection pain/ |
| **Follow up** | N/A | N/A | N/A | N/A | N/A | N/A |

**Supplementary Table 3**

| **STUDY** | **STUDY DESIGN** | **TOTAL NO OF PATIENTS** | **MALE (%)** | | **Age**  **Mean (±SD)** | | **BMI (kg/m2) Mean (±SD)** | | **ASA Score**  **1/2/3/4** | | **OPERATION TIME, MEAN SD** | |
| --- | --- | --- | --- | --- | --- | --- | --- | --- | --- | --- | --- | --- |
|  |  |  | **CIPROFOL** | **PROPOFOL** | **CIPROFOL** | **PROPOFOL** | **CIPROFOL** | **PROPOFOL** | **CIPROFOL** | **PROPOFOL** | **CIPROFOL** | **PROPOFOL** |
| Zeng Y 2022 | multicenter, randomized, open-label, propofol-controlled phase 2 clinical trial | 40 | 11 (36.7) | 3 (30.0) | 42.5 ± 10.3 | 46.4 ± 11.2 | 23.7 ± 3.0 | 23.6 ± 3.6 | 16/14/0/0 | 4/6/0/0 | 105.3 ± 62.6 | 76.7 ± 36.1 |
| Wang X 2022 | phase 3, multicenter, randomized, double-blind, comparative study | 176 | 32 | 31 | 38.5 (12.1) | 41.1 (11.1) | 23.3 (2.9) | 23.3 (3.1) | 51/37/0/0 | 48/40/0/0 | **-** | **-** |
| Qin K 2022 | prospective, randomized, single-blind study | 105 | 18 (34.6) | 18 (34.0) | 39.00±10.10 | 41.25±10.63 | 23.38±3.33 | 22.63±2.38 | 0/0/42/10 | 0/0/44/9 | 175.88±42.22 | 165.49±34.27 |
| Chen-Ben Zhen 2022 | prospective, double-blind, single-center study | 120 | - | - | 33.9±9.1 | 33.8±9.6 | 22.2±3.2 | 21.4±2.8 | 32/28/0/0 | 34/26/0/0 | 55.2±20.5 | 51.4±23.1 |
| Liang Peng 2023 | multicenter, single-blinded, propofol-controlled, randomized, phase 3 trial | 128 | 23 (26.7) | 10 (23.8) | 38.5 ± 10.1 | 40.5 ± 10.1 | 23.3 ± 2.8 | 23.3 ± 3.0 | 48/38/0/0 | 22/20/0/0 | 94.6 ± 39.2 | 93.6 ± 35.8 |
| Man Yan 2023 | randomized double-blind controlled study | 128 | - | - | 42.2±9.46 | 44.1±9.4 | 22.8±2.2 | 23.3±2.6 | 18/46/0/0 | 14/50/0/0 | **-** | 79.8 ± 57 |

**Supplementary table 4**

| OUTCOME | EFFECT SIZE (RR OR WMD) | 95% CI | p-VALUE |
| --- | --- | --- | --- |
| **Total adverse events (induction)**  0.4mg ciprofol  0.5 mg ciprofol | **RR: 0.76**  0.93  0.61 | **0.46,1.25**  0.85,1.01  0.49,1.25 | **0.28**  0.10  <0.0001 |
| **Tachycardia**  0.4mg cipro vs propo (induction)  0.5mg cipro vs propo (induction)  o.4mg ciprofol (induction and maintenance) | **RR: 0.24**  0.24  3.00  0.03 | **0.0040.04,1.51**  0.04,1.45  0.12,72.29  0.00,0.45 | **0.13**  0.12  0.50  0.01 |
| **Rash**  0.4mg cipro vs propo (induction)  0.4mg ciprofol (induction and maintenance) | **RR: 1.33**  1.48  1.26 | **0.22,8.25**  0.06,35.64  0.14,11.68 | **0.76**  0.81  0.84 |
| **Prolonged QT interval** | **RR: 0.43** | **0.05,3.95** | **0.45** |
| **Pain on injection site (induction)**  0.4mg ciprofol  0.5mg ciprofol | **RR: 0.16**  0.26  0.02 | **0.06,0.44**  0.13,0.52  0.00,0.14 | **0.0003**  0.09  <0.0001 |
| **Hypoxia**  0.4mg cipro vs propo (induction)  0.4mg cipro vs propo (indcution and maintenance) | **RR: 1.56**  1.80  1.06 | **0.31,7.94**  0.27,12.09  0.05,24.25 | **0.59**  0.55  0.97 |
| **Hypotension**  0.4mg cipro vs propo (induction)  0.5mg cipro vs propo (induction)  0.4mg (induction and maintenance) | **RR: 0.74**  0.67  0.69  0.71 | **0.53,1.02**  0.35,1.29  0.48,1.01  0.35,1.46 | **0.06**  0.23  0.06  0.35 |
| **Hypertension (induction)**  0.4mg cipro vs propo  0.5mg cipro vs propo | **RR: 1.61**  1.36  2.00 | **0.54,4.86**  0.31,5.95  0.38,10.54 | **0.39**  0.68  0.41 |
| **CTCAE Severity Grading (grade 1)**  0.4mg cipro vs propo (induction)  0.mg cipro vs propo (induction and maintenance) | **RR: 1.21**  1.37  1.00 | **0.89**  1.10,1.70  0.70,1.43 | **0.23**  0.005  1.00 |
| [**CTCAE Severity Grading (Grade 2)**](https://drive.google.com/open?id=1X1QfKe4y03x3Qw_qZp-BFTX93ArC2vQc&usp=drive_copy)  0.4mg cipro vs propo (induction)  0.4mg cipro vs propo (induction and maintenance) | **RR: 0.95**  0.94  0.96 | **0.80,1.14**  0.73,1.22  0.75,1.24 | **0.60**  0.65  0.77 |
| **Bradycardia**  0.4mg cipro vs propo (induction)  0.5mg cipro vs propo (induction)  0.4mg cipro vs propo (induction and maintenance) | **RR: 0.81**  0.89  1.17  0.74 | **0.58,1.14**  0.47,1,70  0.41,3.28  0.45,1.22 | **0.23**  0.72  0.77  0.24 |
| [**Any treatment-emergent adverse events**](https://drive.google.com/open?id=1CJrtClNPL2dgdg1tOuD_dU-IMvEQcYqO&usp=drive_copy)  0.4mg cipro vs propo (induction)  0.4mg cipro vs propo (induction and maintenance) | **RR: 1.07**  1.05  1.14 | **0.96,1.19**  0.94,1.19  0.90,1.45 | **0.21**  0.38  0.29 |
| [**0.4 mg Number of patients who maintained BIS between at 40 - 60 (min)**](https://drive.google.com/open?id=1GmbdE1okwtR-edDxKOLuHUj4oY2AvG-N&usp=drive_copy) | **RR: 1.31** | **0.59,2.90** | **0.51** |
| [**0.4 mg elevated AST (induction and Maintenance)**](https://drive.google.com/open?id=1aDt4e2l2ltY6i6gwWU0yIM8hgq6r1ZeB&usp=drive_copy) | **RR: 0.69** | **0.09,5.40** | **0.72** |


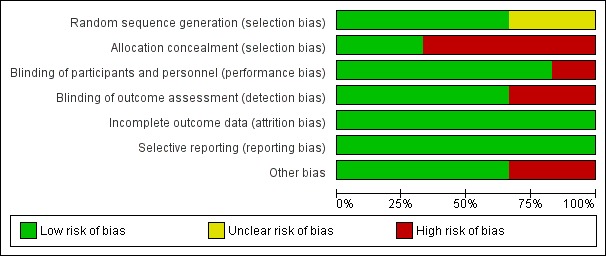
**Figure 1**A


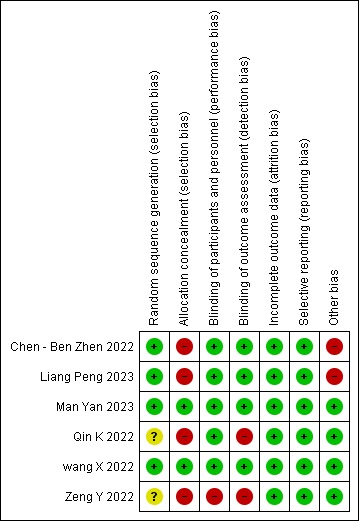


**Figure** 1B


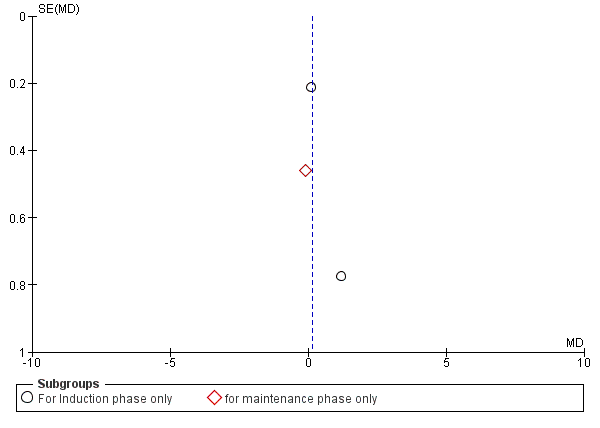


Figure 2


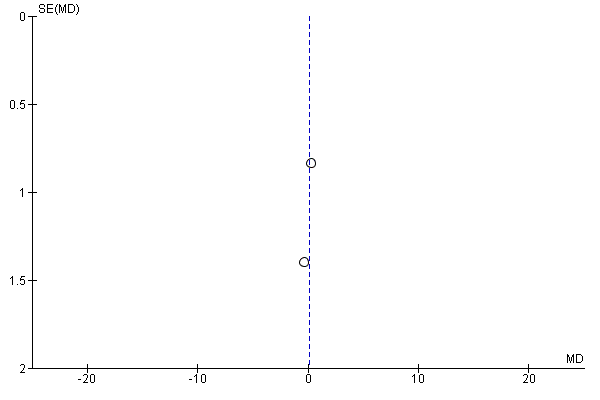


Figure 3


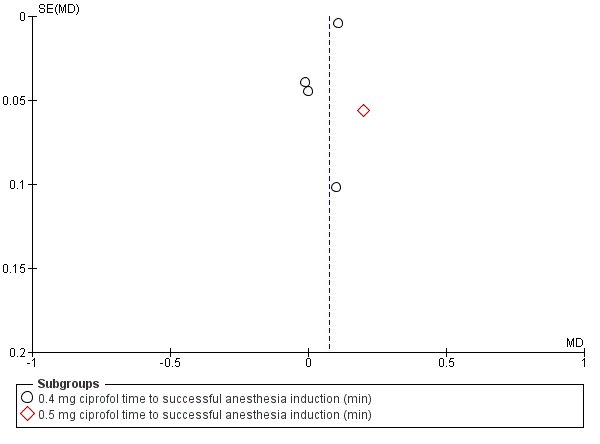


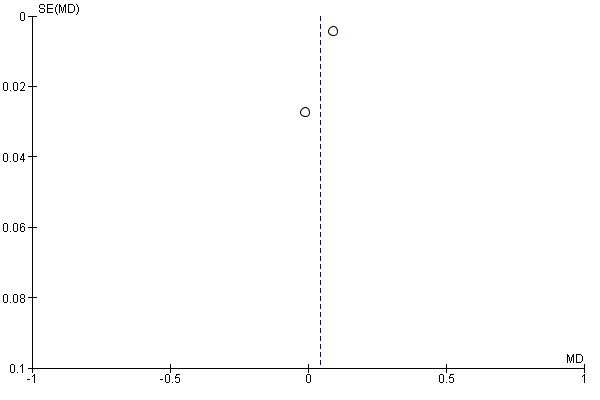
Figure 4

Figure 5


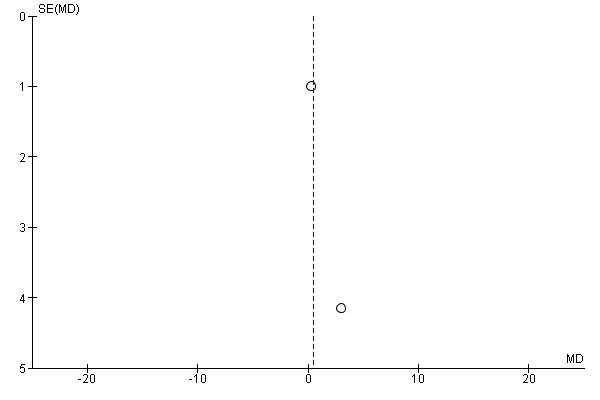


Figure 6


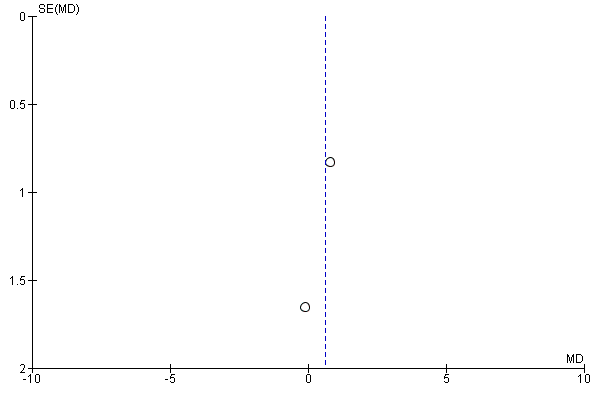


Figure 7


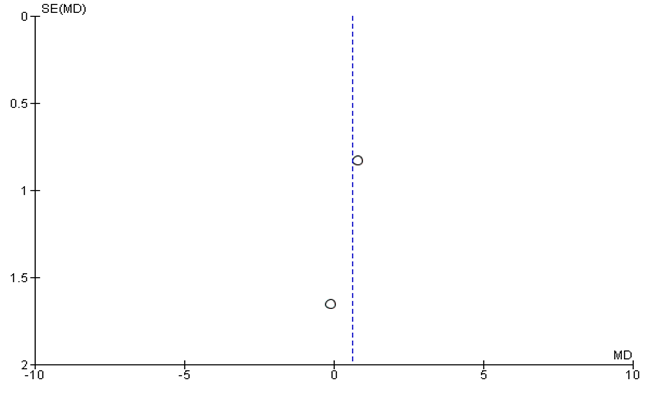


Figure 8
